# Magnitude of undernutrition in children aged 2 to 4 years using CIAF and conventional indices in the slums of Mumbai city

**DOI:** 10.1186/s41043-015-0017-x

**Published:** 2015-07-10

**Authors:** Mitravinda S. Savanur, Padmini S. Ghugre

**Affiliations:** Department of Food Science and Nutrition, Sir Vithaldas Vihar, S.N.D.T. Women’s University, Juhu Road, Mumbai, 400049 Maharashtra India

**Keywords:** CIAF, Underweight, Stunting, Wasting, Nutritional status, Children, India

## Abstract

Conventional indicators – weight-for-age, height-for-age, weight-for-height and mid-upper arm circumference (MUAC) reflect different facets of the nutritional status. Weight-for-age is the most commonly used indicator. When used individually or in combination, conventional indices fail to depict the overall magnitude of undernutrition in the population. Composite Index of Anthropometric Failure (CIAF) is an alternative classification system which attempts to fill this lacuna. Thus, we undertook this study with the objective to compare the prevalence of undernutrition using CIAF and the conventional indices. We included 634 children aged between 2 to 4 years from *anganwadis* located in three areas of Mumbai. Weight, height and MUAC measurements were taken. Z scores were computed for weight-for-age (WAZ), height-for-age (HAZ) and weight-for-height (WHZ) using WHO Anthro software. Children were classified as per the conventional indices and CIAF. The prevalence of underweight, stunting and wasting was 35.7 %, 33.8 % and 18.5 % respectively. None of the children had MUAC < 11.5 cm. About 1 % of the children were moderately wasted according to MUAC. As per CIAF, 47.8 % children were undernourished. According to CIAF, one-third of the undernourished children had single anthropometric failure while half of them had dual failure and 17.1 % had multiple failures. When compared with the conventional indices, CIAF could recognize 12.1 %, 14.0 %, 29.3 % and 46.7 % more undernourished children than WAZ, HAZ, WHZ and MUAC respectively. In conclusion, CIAF is seen to have many advantages over the conventional indices. CIAF is useful in assessing the overall magnitude of undernutrition and identifying children with multiple anthropometric failures. It also recognizes more undernourished children than all the conventional indices. Therefore, CIAF should be used more widely as a tool for nutritional assessment particularly in developing countries where the burden of undernutrition is high.

## Background

Undernutrition among children under five years is traditionally assessed using anthropometric indices such as – weight-for-age, height-for-age, weight-for-height and mid-upper arm circumference (MUAC). Stunting or low height-for-age is an indicator of chronic undernutrition which is manifested as poor skeletal growth. Low weight-for-height reflects wasting or acute undernutrition with loss of lean as well as fat mass [1]. On the other hand, low MUAC (<11.5 cm) is not only suggestive of severe wasting or severe acute malnutrition but also indicative of morbidity and risk of mortality [2, 3]. Underweight or low weight-for-age, on the other hand, is indicative of both acute and chronic undernutrition [1]. These indices reflect different facets of undernutrition. Although underweight, stunting and wasting reflect different facets of undernutrition, they are not mutually exclusive categories. For instance, a child who is found to be stunted can also be underweight and wasted at the same time. Hence, a sum of the children who are underweight, stunted and wasted in a group does not reveal the overall number of undernourished children in a population. The conventional indices therefore fail to provide the overall prevalence of undernutrition in a group.

There is another concern with the use of conventional indices. Weight-for-age is most commonly used to assess the nutritional status. This may be because underweight indicates both acute and chronic undernutrition. However, underweight is not the summation of children who are wasted and stunted. As a result, we might tend to miss out on children who are stunted and wasted if underweight is used as a sole indicator of nutritional status.

In the year 2000, Swedish Economist Prof. Peter Svedberg suggested an alternative measure to assess the overall magnitude of undernutrition – Composite Index of Anthropometric Failure (CIAF). CIAF identifies seven groups of children including those without any form of anthropometric failure (Table 1). A summation of the groups B, C, D, E, F and Y gives the total magnitude of undernutrition. At the same time, it can be useful in detecting multiple anthropometric failures [4].

**Table 1:** Categories of the composite index of anthropometric failure (CIAF)

Group	Description of the group	Definition
A	No anthropometric failure	Normal WAZ, HAZ and WHZ
B	Wasting only	WHZ < -2SD but normal WAZ and HAZ
C	Wasting and underweight	WHZ and WAZ < -2 SD but normal HAZ
D	Wasting, underweight and stunting	WHZ, WAZ and HAZ < -2 SD
E	Stunting and underweight	HAZ and WAZ < -2 SD but normal WHZ
F	Stunting only	HAZ < -2 SD but normal WAZ and WHZ
Y	Underweight only	WAZ < -2 SD but normal HAZ and WHZ

Investigators in Kenya, China and Bangladesh have used CIAF to assess the extent of undernutrition [5–7]. In Kenya, Berger et al estimated the prevalence of undernutrition among children with HIV/AIDS [5]. Khan et al and Pei et al also identified the sociodemographic factors determining overall undernutrition [7, 6]. Nandy and Miranda have used the national data from seven developing countries to calculate the CIAF and compare it with prevalence of underweight in the same areas [8].

In India, Nandy *et al* was the first to use the concept of CIAF on the data of 1998 – 99 National Family Health Survey – 2 (NFHS - 2) [1]. Thereafter most of the Indian studies that have used CIAF have been conducted in rural or tribal areas of West Bengal [9–15]. Only two studies so far have used CIAF in an urban setting i.e. in Coimbatore, Tamil Nadu and Bankura town, West Bengal [16, 17].

In Maharashtra state, the prevalence of underweight, stunting and wasting in under-five children in rural and urban areas was 37 %, 46 % and 17 % respectively. The nutritional status of under-five children in Mumbai was reportedly worse than the other urban areas in the state. The prevalence of underweight and stunting was 40 % and 14 % higher respectively in the slum than the non-slum areas of Mumbai [18]. The overall extent of undernutrition in the children belonging to the slums in Mumbai remains unknown.

We therefore conducted the present study with an objective to compare the prevalence of undernutrition by using CIAF and the conventional indices.

## Methods

The study was approved by Independent Ethics Committee (IEC no 09122), Navi Mumbai, Maharashtra, India.

### Study design

**Fig. 1:**
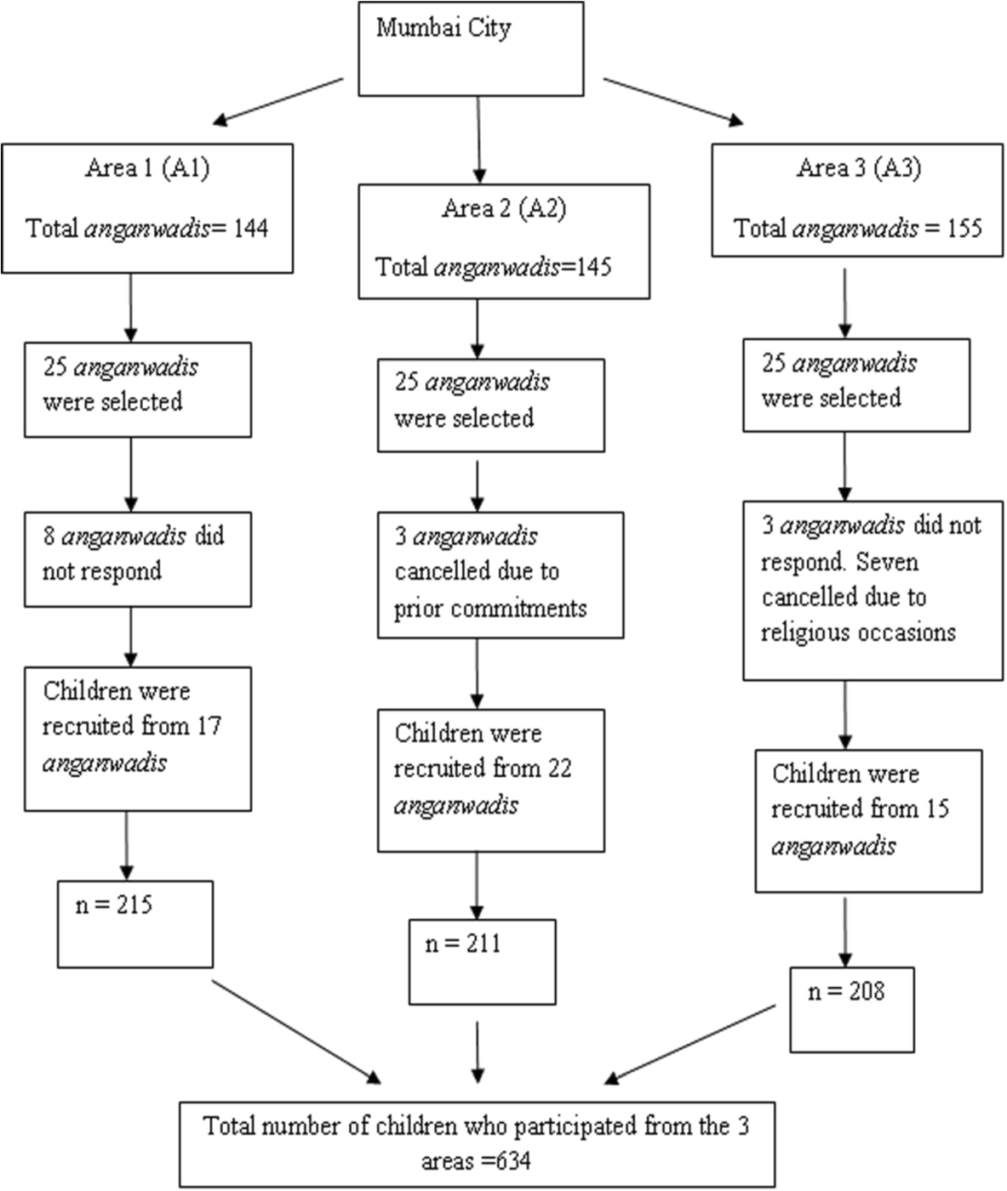
Selection of Participants from Mumbai City

This cross-sectional study was carried out from July, 2013 to January, 2014 in the slums of Mumbai city, Maharashtra, India. We undertook the study in three urban slum areas located in the western suburbs of the city. Each of these areas had 140 to 150 *anganwadis* each. *Anganwadi* is a child-care and mother-care centre which is run by the Integrated Child Development Service (ICDS) in India. We obtained a list of *anganwadis* in each of these areas from the respective Child Development Project Officers (CDPO). From this list, every sixth *anganwadi* was selected by simple random sampling. Thus, from every area, we selected 25 *anganwadis* each (Fig. 1). Participants: Six hundred and thirty four children aged between 2 to 4 years participated in the study. The inclusion and exclusion criteria for including the children in this study were.

### Inclusion criteria

Children who (i) were beneficiaries of the *anganwadi,* (ii) had authentic records of their date of birth and (iii) had completed 24 months of age and were less than or equal to 48 months were included in the study. Age was calculated on the basis of their date of birth to the nearest one month.

### Exclusion criteria

Children who were – (i) suffering from any chronic illness that influenced their nutritional status, (ii) born with congenital anomalies, (iii) born extremely premature (<28 weeks of gestational age).

Parents and/or guardians of the children were explained about the study procedure in the local language and an informed consent was obtained from them.

### Assessment of nutritional status

Weight and height measurements of all the children were taken using standard procedure [19]. Children were weighed on a digital weighing scale (Dr Gene Health and Wellness; Model no: MS8270) with an accuracy of 0.1 kg. Height was measured using a non-extensible, flexible measuring tape with an accuracy of 0.1 cm which was calibrated against the standard anthropometric scale. Based on these measurements, weight-for-age Z scores (WAZ), height-for-age Z scores (HAZ) and weight-for-height Z scores (WHZ) were computed. MUAC was measured using a non-extensible, flexible measuring tape at the mid-point between the acromion and olecranon processes of the child’s arm flexed at 90° angle. The child was then made to relax the arm so that it hangs just away from the side of the body and the circumference was measured at the mid-point with an accuracy of 0.1 cm [20].

Children with WAZ, HAZ and WHZ scores between – 3.0 to – 2.0 SD were classified as moderately underweight, stunted and wasted respectively. Those with WAZ, HAZ and WHZ scores were < -3.0 SD were classified as severely underweight, stunted and wasted respectively. And, those children who had Z scores > -2.0 SD were classified as ‘normal’ [21]. MUAC < 11.5 cm and between 11.5 to 12.5 cm were classified as severe acute malnutrition (SAM) and moderate acute malnutrition (MAM) respectively. Those with MUAC > 12.5 cm were considered ‘normal’ [3]. Further, children were also classified using CIAF [1].

### Statistical analysis

WAZ, HAZ and WHZ scores were computed using WHO Anthro software version 3.2.2. The data was analyzed using SPSS version 20. For WAZ, HAZ, WHZ and MUAC one-way ANOVA was used to analyze the difference in the mean Z scores between boys and girls. Chi-square tests were performed to study the age-wise and gender-wise differences in the prevalence of undernutrition.

## Results

There were 53.6 % (n = 340) boys and 46.4 % (n = 294) girls in the study. The mean age of the children was 3.1 ± 0.8 years. The mean anthropometric measurements of children are presented in Table 2. Boys weighed significantly more (F = 12.039, p = 0.001) and had a significantly higher MUAC (F = 4.151, p = 0.042) than the girls. However no statistical differences were seen between boys and girls for the other anthropometric parameters.

**Table 2:** Mean anthropometric measurements of boys and girls (n = 634)

Parameter	Boys (n = 340)	Girls (n = 294)	F	*P* value
Weight (kg)	11.9 ± 1.8	11.4 ± 1.8	12.039	0.001
Height (cm)	90.1 ± 7.2	89.2 ± 7.0	2.427	0.120
WAZ	-1.6 ± 1.0	-1.7 ± 0.9	2.194	0.139
HAZ	-1.5 ± 1.2	-1.6 ± 1.1	0.921	0.337
WHZ	-1.1 ± 1.0	-1.1 ± 0.9	0.000	0.987
MUAC (cm)	14.9 ± 1.1	14.8 ± 1.1	4.151	0.042

*WAZ* Weight-for-age Z score, *HAZ* Height-for-age Z score, *WHZ* Weight-for-height Z score, *MUAC* Mid-upper arm circumference

### Nutritional Status

#### Using conventional indices

Children were classified as per WHO Growth Standards [3, 21]. One hundred and seventy-six (27.8 %) children were moderately underweight and 50 (7.9 %) were severely underweight (Table 3). One hundred and forty-three (22.6 %) children and 71(11.2 %) were moderately and severely stunted respectively. One hundred and four (16.4 %) children and 13 (2.1 %) children had moderate and severe wasting respectively. None of the children had a MUAC < 11.5 cm. Seven children (1.1 %) had MAM according to MUAC. There were no significant differences were observed in the prevalence rates in boys and girls. Similarly, no statistical differences were seen in the prevalence of underweight and stunting across the age groups. However, significantly higher number of children in the age group of 3 to 4 years were moderately wasted than children aged 2 to 3 years of age (20.3 % v 12 %; *χ*2 = 7.983, p = 0.018).

**Table 3:** Prevalence of undernutrition according to the conventional indices

Indicator	Classification	Age (years)		Sex		Total % (n)
		2 to 3 % (n)	3 to 4 % (n)	Boys % (n)	Girls % (n)	
WAZ (Underweight)	Normal	64.5 (209)	64.2 (199)	66.8 (227)	61.6 (181)	64.4 (408)
	Moderate underweight	29.6 (96)	25.8 (80)	25.6 (87)	30.3 (89)	27.8 (176)
	Severe underweight	5.9 (19)	10.0 (31)	7.6 (26)	8.2 (24)	7.9 (50)
	*χ*2	4.273		1.962		
	p	0.118		0.375		
HAZ (Stunting)	Normal	66.0 (214)	66.5 (206)	67.6 (230)	64.6 (190)	66.2 (420)
	Moderate stunting	22.2 (72)	22.9 (71)	20.9 (71)	24.5 (72)	22.6 (143)
	Severe stunting	11.7 (38)	10.6 (33)	11.5 (39)	10.9 (32)	11.2 (71)
	*χ*2	0.202		1.175		
	p	0.904		0.556		
WHZ (Wasting)	Normal	85.8 (278)	77.1 (239)	79.7 (271)	83.7 (246)	81.5 (517)
	Moderate wasting	12.7 (41)	20.3 (63)	17.9 (61)	14.6 (43)	16.4 (104)
	Severe wasting	1.5 (5)	2.6 (8)	2.4 (8)	1.7 (5)	2.1 (13)
	*χ*2	7.983		1.688		
	p	0.018		0.430		
MUAC (Acute malnutrition)	Normal (>12.5 cm)	99.1 (321)	98.7 (306)	98.8 (336)	99.0 (291)	98.9 (627)
	MAM (11.5 to 12.5 cm)	0.9 (3)	1.3 (4)	1.2 (4)	1.0 (3)	1.1 (7)
	*χ*2	0.193		0.035		
	p	0.720		1.000		

*WAZ* Weight-for-age Z score, *HAZ* Height-for-age Z score, *WHZ* Weight-for-height Z score, *MUAC* Mid-upper arm circumference

#### Using composite index of anthropometric failure

According to CIAF classification, 331 children (52.2 %) were well nourished (Table 4). Nearly half the children i.e. 303 (47.8 %) were undernourished. Of all the undernourished children, 101 children (33.3 %) suffered from single anthropometric failure (Group – B, F and Y). Almost half the undernourished children (49.5 %) suffered from dual anthropometric failure (Group – C and E) while fifty-two children i.e. 17.1 % experienced multiple failures i.e. they were underweight, stunted and wasted at the same time. No age-wise (*χ*2 = 11.516, p = 0.074) and sex-wise (*χ*2 = 10.864, p = 0.093) differences were noted in the prevalence of single, dual and multiple anthropometric failures.

**Table 4:** Prevalence of undernutrition according to CIAF

Group	Description of the group	Age (years)		Sex		Total % (n)
		2 to 3 % (n)	3 to 4 % (n)	Boys % (n)	Girls % (n)	
A	No anthropometric failure	53.7 (174)	50.6 (157)	54.1 (184)	50.0 (147)	52.2 (331)
B	Wasting only	1.2 (4)	4.2 (13)	3.8 (13)	1.3 (4)	2.6 (17)
C	Wasting and underweight	6.2 (20)	8.7 (27)	6.7 (23)	8.5 (25)	7.5 (48)
D	Wasting, underweight and stunting	6.8 (22)	9.7 (30)	9.7 (33)	6.4 (19)	8.2 (52)
E	Stunting and underweight	17.6 (57)	14.8 (46)	13.8 (47)	18.7 (55)	16.1 (102)
F	Stunting only	9.6 (31)	9.4 (29)	8.8 (30)	10.2 (30)	9.4 (60)
Y	Underweight only	4.9 (16)	2.6 (8)	2.9 (10)	4.76 (14)	3.7 (24)
	Total anthropometric failure (B + C + D + E + F + Y)	46.3 (150)	49.4 (153)	45.8 (156)	50.0 (147)	47.8 (303)
	*χ*2	11.516		10.864		
	p	0.074		0.093		

#### CIAF v Conventional indices of undernutrition

CIAF could identify more undernourished children than the conventional indices. The prevalence of undernutrition according to CIAF was 47.8 %. On the other hand, using conventional indices, 35.7 % were underweight, 33.8 % were stunted and 18.5 % were wasted. Thus, CIAF could recognize 12.1 %, 14.0 % and 29.3 % more undernourished children than WAZ, HAZ and WHZ respectively. CIAF could recognize 46.7 % more children as undernourished than MUAC. Among all the children classified as well nourished by MUAC, 16.1 % had single anthropometic failure while 23.4 % and 7.7 % suffered from dual and multiple failures respectively (Table 5).

**Table 5:** Distribution of children in MUAC categories across the CIAF

CIAF Classification	MUAC Categories % (n)	
	11.5 to 12.5 cm	>12.5 cm
	(n = 7)	(n = 627)
No failure	0	52.8 (329)
Wasting only	0	2.7 (17)
Wasting and underweight	14.3 (1)	7.3 (46)
Wasting, underweight and stunting	57.1 (4)	7.7 (48)
Stunting and underweight	28.6 (2)	16.1 (101)
Stunting only	0	9.6 (60)
Underweight only	0	3.8 (24)

## Discussion

The present study compared the prevalence of undernutrition by using CIAF and the conventional indices. The prevalence of undernutrition according to CIAF was 47.8 %. On the other hand, according to the conventional indices 35.7 %, 33.8 % and 18.5 % children were underweight, stunted and wasted respectively. Also, about 1 % children were classified as MAM according to MUAC.

Some researchers have used CIAF to assess the prevalence of undernutrition. Nandy and Miranda [8] computed the CIAF using the national data from 1998 to 2001 of seven developing countries - India, Ethopia, Nepal, Tanzania, Zimbabwe, Bolivia and Peru. The prevalence rates in Peru (23.3 %), Bolivia (26.6 %) and Zimbabwe (35.8 %) were lower than that observed our study while in Nepal (56.5 %) and Ethopia (58 %) rates were much higher. Recent reports indicate that 21.4 % children were undernourished in rural China [6] while in rural Bangladesh the prevalence was 58.7 % [7]. On the other hand, the rates were slightly lower in urban Bangladesh (47.9 %) and were comparable to that observed in our study [7].

In India, a total of 59.8 % children were reported to be undernourished using the NFHS – 2 data collected during 1998 – 99 [8]. Other studies in India have been mainly conducted in rural and tribal West Bengal wherein the prevalence rates ranged from 50.2 to 73.0 % which were much higher than those observed in our study [9–14]. Similarly, 59.6 % were reported to be undernourished in rural Wardha, Maharashtra state [22]. Factors associated with undernutrition in these rural and tribal areas were reported to be birth order, low birth weight, breastfeeding practices and mother’s education [15]. Only two studies have been reported from urban areas of India, one from Tamil Nadu [16] and another from West Bengal [17]. The prevalence rates in these studies were higher than our study. These differences can be possibly attributed to various factors such as socioeconomic status, educational level, living conditions, maternal health, birth weight, feeding practices and rates of infections. However, we have not studied the factors influencing the overall prevalence of undernutrition as a part of this paper.

In the studies where CIAF and conventional indices were used together, CIAF identified more undernourished children than the latter [8, 14, 16, 23]. Nandy and Miranda [8] found that the use of weight-for-age underestimated the prevalence by 9.7 to 21.7 % as compared to CIAF. Similar findings have been noted by some other researchers in India [14, 16, 23]. However, these workers compared CIAF with only WAZ. We compared CIAF with WAZ, HAZ, WHZ and MUAC. WAZ underestimated the prevalence by 12.1 %, HAZ by 14.0 % and WHZ by 29.3 % as compared to CIAF. Also, MUAC identified 1.1 % children as MAM but failed to identify 46.7 % children who had varying degrees of undernutrition. Thus the conventional indices underestimated the prevalence of undernutrition and erroneously classified a sizeable section of the population as ‘normal’.

Further, conventional indicators do not give a holistic picture of the nutritional status of a population. For instance, by using conventional indices we can find out how many children are underweight. But, there could be some children who are wasted as well as underweight. Such groups of children suffering from two or more anthropometric failures remain unidentified when conventional measures are used. In view of this, Svedberg added three categories to the existing conventional indicators (Group C: wasting and underweight, D: underweight, stunting and wasting and E: underweight and stunting) [4]. In this study, only 33 % of the undernourished children exhibited single anthropometric failure while the remaining had either dual or multiple anthropometric failures which was not revealed with the use of conventional indices. These children exhibiting multiple anthropometric failures will require regular counseling and monitoring than those having single anthopometric failure.

In India, ICDS is the national programme that aims at reducing undernutrition and improving the nutritional status of children up to six years of age. Presently, the ICDS uses weight-for-age and MUAC as the indicators of nutritional status. WAZ identifies underweight children while MUAC recognizes those with extreme wasting. Stunted and moderately/severely wasted children are not identified as undernourished. As a result, a significant proportion of children suffering from dual or multiple failures are missed out. These vulnerable children fail to receive adequate attention and appropriate counseling from the *anganwadi* workers. This can further increase the burden of undernutrition and risk for morbidity. Thus, with the use of CIAF all the undernourished children can be identified.

The ICDS routinely weighs the children. If height is also measured then CIAF can be easily used. Using CIAF will not incur any extra financial burden on the government machineries. The use of CIAF will not only help in assessment but also in monitoring the growth of these children suffering from various degrees of anthropometric failure. Parents can be sensitized and appropriately counseled regarding their child’s nutritional status. Such steps can help reduce the overall burden of undernutrition and also pave the path towards attaining the Millennium Development Goals (MDGs).

The strength of the present study is that this is the first study to have used CIAF to estimate the overall prevalence of undernutrition in the slums of Mumbai. Also, the study compares the prevalence rates of CIAF with all the conventional indices including MUAC. The limitation of our study was that it was carried out in three slum areas of Mumbai city. It would have been worthwhile if similar data was gathered from the non-slum areas where people across the socioeconomic spectrum reside. Also, we used only anthropometric indices and did not include any biochemical parameters for the assessment of nutritional status.

## Conclusion

CIAF is a useful tool which provides a holistic picture of the overall prevalence of undernutrition. Conventional indicators when used individually grossly underestimate the extent of undernutrition. ICDS should thus, consider using CIAF at the national level as a step towards reducing the overall burden of undernutrion in children.
